# Supplementary material for: Identification of photoperiod-induced specific miRNAs in the adrenal glands of Sunite sheep (Ovis aries)
Source: Front Vet Sci. 2022 Jul 22;9:888207. doi: 10.3389/fvets.2022.888207 (PMC9354845; doi:10.3389/fvets.2022.888207)
Supplement: Supplementary file 1 [file Data_Sheet_1.ZIP › Supplementary file/miRDeep2 file.zip!/miRDeep2 file/Know_Structure/SP42A1/oar-mir-99a.pdf]

miRBase precursor : oar-mir-99a
Total read count : 553296
oar-miR-99a read count : 552758
remaining reads : 538

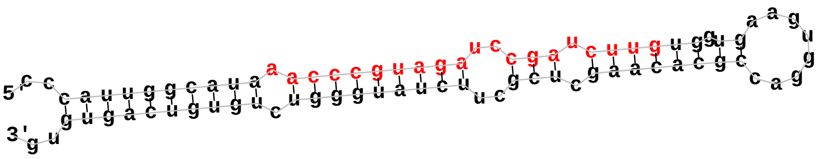

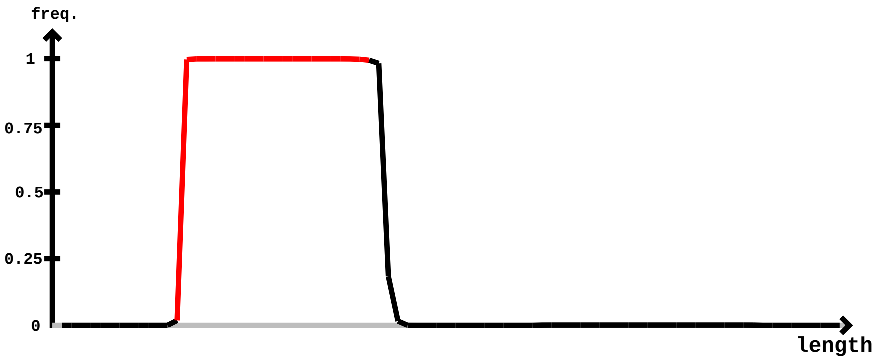

oar-miR-99a

```
5'- cccauuggcauaaacccguagauccgaucuuguggugaaguggaccgcacaagcucgcuucuaugggucugugucagugug  -3'   exp
    ..((((((((((.(((((((((..(((.((((((.((........)))))))).)))..))))))))).))))))))))..    reads   mm   sample
    .........auaaacccguagauccgaucuugu................................................    1       0    seq
    ..........uaaacccguagauccgaucuugu................................................    3       0    seq
    ...........aaacccguagauccgauc....................................................    2       0    seq
    ...........aaacccguagauccgaucu...................................................    10      0    seq
    ...........aaacccguagauccgaucuu..................................................    40      0    seq
    ...........aaacccguagauccgaucuug.................................................    84      0    seq
    ...........aaacccguagauccgaAcuug.................................................    1       1    seq
    ...........aaacccguagauccgaucuugu................................................    9833    0    seq
    ...........aaacccguagauccgaAcuugug...............................................    1       1    seq
    ...........aaacccguagauccgaucuugug...............................................    196     0    seq
    ...........aaacccguagauccgaucuugugg..............................................    10      0    seq
    ............aacccguagauccgauc....................................................    106     0    seq
    ............aacccguagauccgaAc....................................................    4       1    seq
    ............aacccguagauccgaAcu...................................................    46      1    seq
    ............aacccguagauccgaucu...................................................    574     0    seq
    ............aacccguagauccgaucuu..................................................    1922    0    seq
    ............aacccguagauccgaAcuu..................................................    108     1    seq
    ............aacccguagauccgaAcuug.................................................    753     1    seq
    ............aacccguagauccgaucuug.................................................    5667    0    seq
    ............aacccguagauccgaAcuugu................................................    13549   1    seq
    ............aacccguagauccgaucuugu................................................    417687  0    seq
    ............aacccguagauccgaAcuugug...............................................    20813   1    seq
    ............aacccguagauccgaucuugug...............................................    71363   0    seq
    ............aacccguagauccgaucuugugg..............................................    8497    0    seq
    ............aacccguagauccgaAcuugugg..............................................    259     1    seq
    ............aacccguagauccgaAcuuguggu.............................................    3       1    seq
    ............aacccguagauccgaucuuguggu.............................................    29      0    seq
    .............acccguagauccgaAcu...................................................    1       1    seq
    .............acccguagauccgaucuu..................................................    1       0    seq
    .............acccguagauccgaucuug.................................................    8       0    seq
    .............acccguagauccgaAcuug.................................................    12      1    seq
    .............acccguagauccgaucuugu................................................    463     0    seq
    .............acccguagauccgaAcuugu................................................    140     1    seq
    .............acccguagauccgaAcuugug...............................................    407     1    seq
    .............acccguagauccgaucuugug...............................................    83      0    seq
```

oar-miR-99a

cccauuggcauaaacccguagauccgaucuuguggugaaguggaccgcacaagcucgcuucuaugggucugugucagugug			
.............acccguagauccgaAcuugugg..	8	1	seq
.............acccguagauccgaucuugugg..	9	0	seq
..............cccguagauccgaucuugu..	8	0	seq
..............cccguagauccgaucuugug...	1	0	seq
..............cccguagauccgaAcuugug...	3	1	seq
...............ccguagauccgaucuugu..	28	0	seq
...............ccguagauccgaAcuugu..	1	1	seq
...............ccguagauccgaucuugug...	1	0	seq
...............ccguagauccgaAcuugug...	3	1	seq
...............ccguagauccgaucuugugg..	1	0	seq
................cguagauccgaucuugu..	15	0	seq
................cguagauccgaAcuugu..	1	1	seq
................cguagauccgaAcuugug...	1	1	seq
................cguagauccgaucuugug...	3	0	seq
....caagcucgcuucuaugggucu...........	6	0	seq
....caagcucgcuucuaugggucug..........	14	0	seq
....caagcucgcuucuaugggucugu.........	499	0	seq
....aagcucgcuucuaugggucug..........	1	0	seq
....aagcucgcuucuaugggucugu.........	17	0	seq
